# 2-Methyl-4-[(4-methyl­phen­yl)amino]­benzoic acid

**DOI:** 10.1107/S2414314625004559

**Published:** 2025-05-30

**Authors:** Fang He, Sihui Long

**Affiliations:** a School of Chemical Engineering and Pharmacy Wuhan Institute of Technology,Wuhan Hubei 430205 People’s Republic of China; University of Aberdeen, United Kingdom

**Keywords:** synthon, hydrogen bond, acid–acid dimer, single-crystal

## Abstract

In the title compound, C_15_H_15_NO_2_, which was prepared using a Buchwald–Hartwig cross-coupling reaction followed by ester hydrolysis, the dihedral angle between the aromatic rings is 42.44 (7)°. In the crystal, the mol­ecules associate to form centrosymmetric acid–acid dimers.

## Abstract 2

The mol­ecules of the title compound form acid–acid homodimers in the crystal structure.

## Graphical abstract

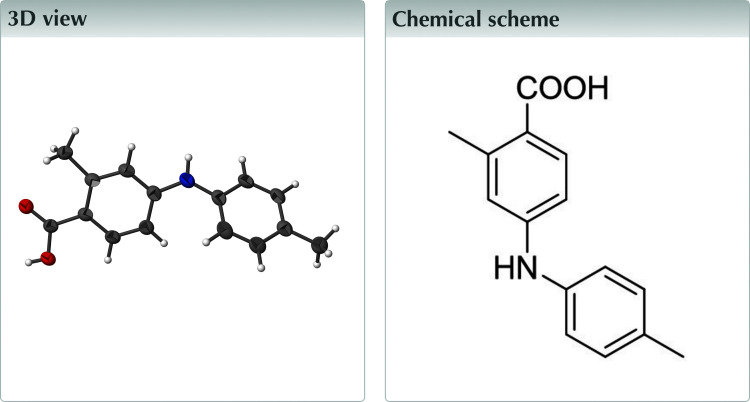

## Structure description

Non-steroidal anti-inflammatory drugs (NSAIDs) are a class of widely used therapeutic drugs in clinical practice for reducing pain and inflammation, and have been shown to protect against many other diseases (Zinellu *et al.*, 2005 ▸), including cancer and cardiac diseases (Bindu *et al.*, 2020 ▸). They are among the most commonly used over-the-counter drugs worldwide (Tsutsumi *et al.*, 2004 ▸). Among the NSAIDs, anthranilic acids are an important branch. For example mefenamic acid can be used to treat mild to moderate postoperative pain and vascular headache, *etc*., and can be rapidly absorbed by the human body after oral administration (Samie *et al.*, 2017 ▸). There are currently three known crystal forms of mefenamic acid (Cimolai, 2013 ▸). As part of our studies in this area, we now report the synthesis and structure of the title compound, (I), which was prepared by a Buchwald–Hartwig cross-coupling reaction between methyl 4-bromo-2-methyl­benzoate and 4-methyl­aniline followed by ester hydrolysis.

**Figure 1:**
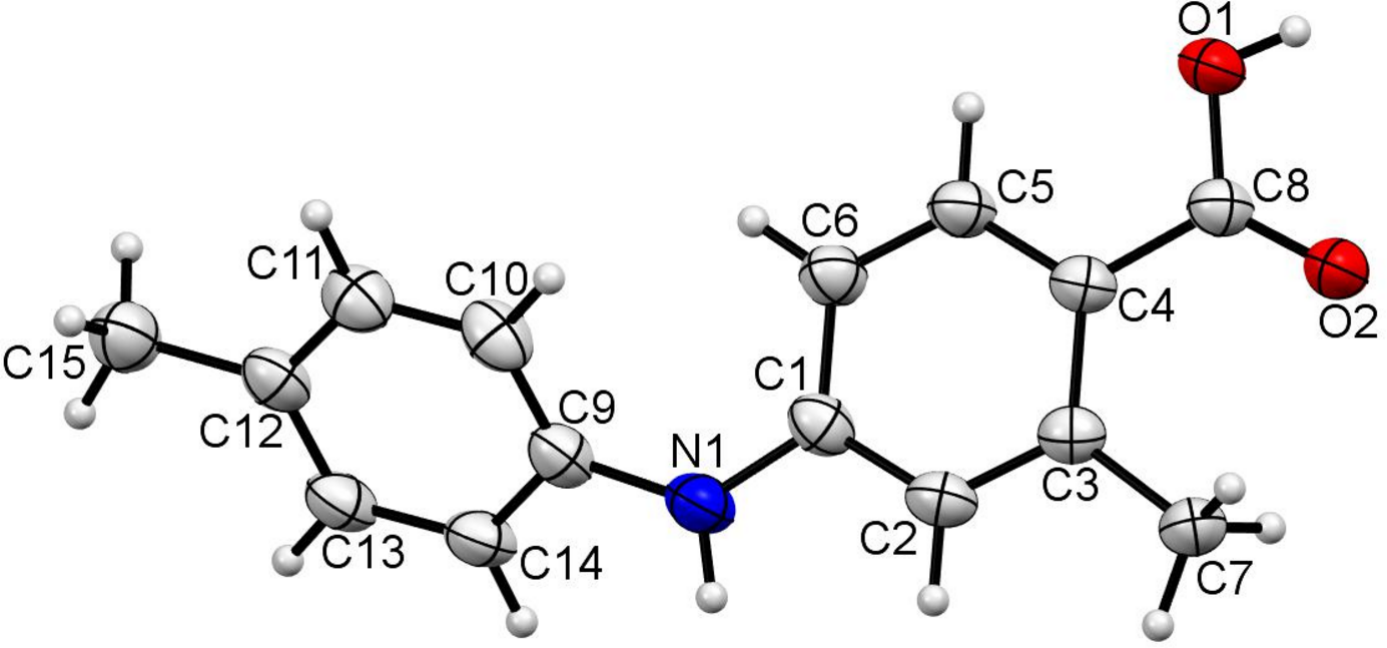
The mol­ecular structure of (I) with displacement ellipsoids drawn at the 50% probability level.

**Figure 2:**
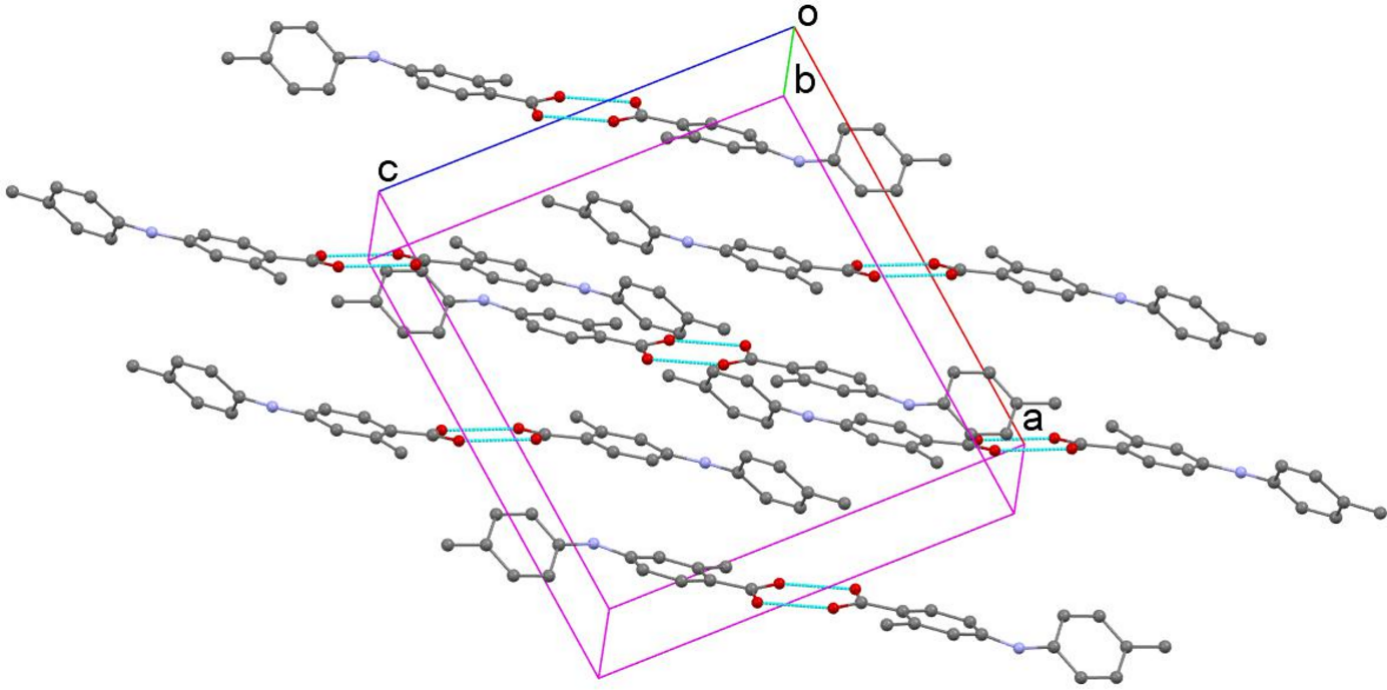
Packing of the mol­ecules of (I) with hydrogen bonds indicated by blue dashed lines (for clarity, H atoms not involved in hydrogen bonding are omitted).

The C1–C6 and C9–C14 aromatic rings in (I) are not coplanar (Fig. 1 ▸) due to steric repulsion: they subtend a dihedral angle of 42.44 (7)°. In the crystal, the mol­ecules form centrosymmetric carb­oxy­lic acid–carb­oxy­lic acid hydrogen-bonded dimers linked by pairwise O—H⋯O hydrogen bonds thereby generating 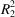
(8) loops (Fig. 2 ▸). Weak pairwise N—H⋯π and C—H⋯O inter­actions also occur (Table 1 ▸).

## Synthesis and crystallization

**Figure 3:**
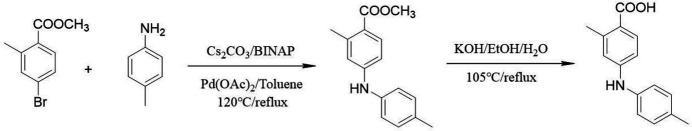
Synthesis scheme for (I).

The title compound was synthesized in two steps using a Buchwald–Hartwig cross-coupling reaction and a hydrolysis reaction (Fig. 3 ▸). The compound was purified by column chromatography. Single crystals were obtained by slowly evaporating an ethyl acetate solution of the compound.

## Refinement

Crystal data, data collection and structure refinement details are summarized in Table 2 ▸.

## Supplementary Material

Crystal structure: contains datablock(s) global, I. DOI: 10.1107/S2414314625004559/hb4493sup1.cif

Structure factors: contains datablock(s) I. DOI: 10.1107/S2414314625004559/hb4493Isup2.hkl

Supporting information file. DOI: 10.1107/S2414314625004559/hb4493Isup3.cml

CCDC reference: 2453294

Additional supporting information:  crystallographic information; 3D view; checkCIF report

## Figures and Tables

**Table 1:** Hydrogen-bond geometry (Å, °)

*D*—H⋯*A*	*D*—H	H⋯*A*	*D*⋯*A*	*D*—H⋯*A*
O1—H1⋯O2^i^	0.84	1.81	2.6450 (16)	175
N1—H1*A*⋯*Cg*2^ii^	0.88	2.93	3.5460 (15)	128
C14—H14⋯O1^iii^	0.95	2.51	3.4368 (18)	165

Symmetry codes: (i) 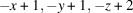; (ii) 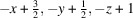; (iii) 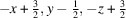.

**Table 2:** Experimental details

Crystal data	
Chemical formula	C_15_H_15_NO_2_
*M*_r_	241.28
Crystal system, space group	Monoclinic, *C*2/*c*
Temperature (K)	150
*a*, *b*, *c* (Å)	16.4180 (5), 9.9205 (3), 15.2137 (5)
β (°)	95.722 (3)
*V* (Å^3^)	2465.58 (12)
*Z*	8
Radiation type	Cu *K*α
μ (mm^−1^)	0.69
Crystal size (mm)	0.44 × 0.32 × 0.21

Data collection	
Diffractometer	ROD, Synergy Custom system, HyPix
Absorption correction	Multi-scan (*CrysAlis PRO*; Rigaku OD, 2024 ▸)
*T*_min_, *T*_max_	0.525, 1.000
No. of measured, independent and observed [*I* > 2σ(*I*)] reflections	8217, 2419, 2168
*R*_int_	0.027
(sin θ/λ)_max_ (Å^−1^)	0.629

Refinement	
*R*[*F*^2^ > 2σ(*F*^2^)], *wR*(*F*^2^), *S*	0.046, 0.133, 1.10
No. of reflections	2419
No. of parameters	167
H-atom treatment	H-atom parameters constrained
Δρ_max_, Δρ_min_ (e Å^−3^)	0.25, −0.21

Computer programs: *CrysAlis PRO* (Rigaku OD, 2024 ▸), *SHELXT* (Sheldrick, 2015*a* ▸a), *SHELXL2018/3* (Sheldrick, 2015*b* ▸) and *OLEX2* (Dolomanov *et al.*, 2009 ▸).

## full crystallographic data

### 2-Methyl-4-[(4-methylphenyl)amino]benzoic acid Crystal data

**Table:**

C_15_H_15_NO_2_	*F*(000) = 1024
*M**_r_* = 241.28	*D*_x_ = 1.300 Mg m^−^^3^
Monoclinic, *C*2/*c*	Cu *K*α radiation, λ = 1.54184 Å
*a* = 16.4180 (5) Å	Cell parameters from 6313 reflections
*b* = 9.9205 (3) Å	θ = 5.2–75.8°
*c* = 15.2137 (5) Å	µ = 0.69 mm^−^^1^
β = 95.722 (3)°	*T* = 150 K
*V* = 2465.58 (12) Å^3^	Block, colourless
*Z* = 8	0.44 × 0.32 × 0.21 mm

### 2-Methyl-4-[(4-methylphenyl)amino]benzoic acid Data collection

**Table:**

ROD, Synergy Custom system, HyPix diffractometer	2419 independent reflections
Radiation source: Rotating-anode X-ray tube, Rigaku (Cu) X-ray Source	2168 reflections with *I* > 2σ(*I*)
Mirror monochromator	*R*_int_ = 0.027
Detector resolution: 10.0000 pixels mm^-1^	θ_max_ = 75.8°, θ_min_ = 5.2°
ω scans	*h* = −20→20
Absorption correction: multi-scan (CrysAlisPro; Rigaku OD, 2024)	*k* = −12→8
*T*_min_ = 0.525, *T*_max_ = 1.000	*l* = −19→18
8217 measured reflections	

### 2-Methyl-4-[(4-methylphenyl)amino]benzoic acid Refinement

**Table:**

Refinement on *F*^2^	Hydrogen site location: inferred from neighbouring sites
Least-squares matrix: full	H-atom parameters constrained
*R*[*F*^2^ > 2σ(*F*^2^)] = 0.046	*w* = 1/[σ^2^(*F*_o_^2^) + (0.0739*P*)^2^ + 1.2032*P*] where *P* = (*F*_o_^2^ + 2*F*_c_^2^)/3
*wR*(*F*^2^) = 0.133	(Δ/σ)_max_ < 0.001
*S* = 1.10	Δρ_max_ = 0.25 e Å^−^^3^
2419 reflections	Δρ_min_ = −0.20 e Å^−^^3^
167 parameters	Extinction correction: *SHELXL2018/3* (Sheldrick, 2015b), Fc^*^=kFc[1+0.001xFc^2^λ^3^/sin(2θ)]^-1/4^
0 restraints	Extinction coefficient: 0.0029 (4)
Primary atom site location: dual	

### 2-Methyl-4-[(4-methylphenyl)amino]benzoic acid Special details

**Table:**

Geometry. All esds (except the esd in the dihedral angle between two l.s. planes) are estimated using the full covariance matrix. The cell esds are taken into account individually in the estimation of esds in distances, angles and torsion angles; correlations between esds in cell parameters are only used when they are defined by crystal symmetry. An approximate (isotropic) treatment of cell esds is used for estimating esds involving l.s. planes.
Refinement. The O-bound H atom was located in a difference map and refined in its as-found relative position. The other H atoms were geometrically placed in idealized locations (C—H = 0.95–0.98, N—H = 0.88 Å) and refined as riding atoms.

### 2-Methyl-4-[(4-methylphenyl)amino]benzoic acid Fractional atomic coordinates and isotropic or equivalent isotropic displacement parameters (Å^2^)

**Table:**

	*x*	*y*	*z*	*U*_iso_*/*U*_eq_	
O1	0.53523 (7)	0.57979 (10)	0.90364 (8)	0.0453 (3)	
H1	0.513945	0.592474	0.950925	0.068*	
O2	0.52812 (7)	0.36584 (11)	0.94716 (7)	0.0467 (3)	
N1	0.74595 (8)	0.32437 (14)	0.61998 (9)	0.0445 (4)	
H1A	0.758886	0.238319	0.619262	0.053*	
C1	0.69439 (8)	0.36056 (15)	0.68229 (10)	0.0369 (4)	
C2	0.65895 (8)	0.25743 (14)	0.72803 (10)	0.0366 (3)	
H2	0.668889	0.166836	0.711902	0.044*	
C3	0.61021 (8)	0.28018 (14)	0.79546 (9)	0.0332 (3)	
C4	0.59475 (8)	0.41564 (14)	0.81851 (9)	0.0335 (3)	
C5	0.62848 (9)	0.51880 (15)	0.77126 (10)	0.0374 (3)	
H5	0.616905	0.609612	0.785434	0.045*	
C6	0.67791 (9)	0.49426 (15)	0.70487 (10)	0.0393 (4)	
H6	0.700533	0.567010	0.674770	0.047*	
C7	0.57618 (9)	0.16081 (14)	0.83972 (10)	0.0397 (4)	
H7A	0.599543	0.157063	0.901512	0.059*	
H7B	0.590199	0.078181	0.809301	0.059*	
H7C	0.516513	0.169078	0.837310	0.059*	
C8	0.54936 (8)	0.45005 (14)	0.89410 (9)	0.0348 (3)	
C9	0.78086 (9)	0.40470 (16)	0.55745 (10)	0.0385 (4)	
C10	0.74147 (9)	0.51372 (17)	0.51438 (10)	0.0436 (4)	
H10	0.688559	0.539547	0.528380	0.052*	
C11	0.77868 (9)	0.58451 (17)	0.45156 (10)	0.0436 (4)	
H11	0.751010	0.659471	0.423548	0.052*	
C12	0.85580 (9)	0.54941 (16)	0.42783 (10)	0.0404 (4)	
C13	0.89357 (9)	0.43812 (16)	0.47018 (11)	0.0427 (4)	
H13	0.945586	0.410170	0.454627	0.051*	
C14	0.85766 (9)	0.36754 (15)	0.53389 (11)	0.0423 (4)	
H14	0.885419	0.292813	0.562098	0.051*	
C15	0.89559 (11)	0.62890 (17)	0.35944 (12)	0.0504 (4)	
H15A	0.910292	0.718655	0.382809	0.076*	
H15B	0.857318	0.637784	0.306056	0.076*	
H15C	0.945053	0.582004	0.344935	0.076*	

### 2-Methyl-4-[(4-methylphenyl)amino]benzoic acid Atomic displacement parameters (Å^2^)

**Table:**

	*U*^11^	*U*^22^	*U*^33^	*U*^12^	*U*^13^	*U*^23^
O1	0.0555 (7)	0.0317 (6)	0.0516 (7)	0.0040 (5)	0.0205 (5)	−0.0048 (4)
O2	0.0583 (7)	0.0346 (6)	0.0506 (7)	0.0008 (5)	0.0217 (5)	−0.0026 (5)
N1	0.0459 (7)	0.0369 (7)	0.0531 (8)	0.0010 (5)	0.0170 (6)	−0.0082 (6)
C1	0.0315 (7)	0.0391 (8)	0.0403 (7)	−0.0008 (6)	0.0050 (6)	−0.0069 (6)
C2	0.0359 (7)	0.0313 (7)	0.0418 (7)	0.0022 (6)	0.0009 (6)	−0.0071 (6)
C3	0.0301 (6)	0.0308 (7)	0.0380 (7)	−0.0001 (5)	−0.0009 (5)	−0.0027 (5)
C4	0.0310 (6)	0.0307 (7)	0.0386 (7)	0.0013 (5)	0.0021 (5)	−0.0039 (5)
C5	0.0399 (7)	0.0296 (7)	0.0429 (8)	0.0010 (6)	0.0055 (6)	−0.0043 (6)
C6	0.0398 (7)	0.0338 (8)	0.0452 (8)	−0.0028 (6)	0.0089 (6)	−0.0041 (6)
C7	0.0473 (8)	0.0302 (7)	0.0420 (8)	0.0003 (6)	0.0069 (6)	−0.0006 (6)
C8	0.0334 (7)	0.0305 (7)	0.0405 (7)	0.0000 (5)	0.0039 (5)	−0.0026 (6)
C9	0.0354 (7)	0.0395 (8)	0.0416 (8)	−0.0024 (6)	0.0081 (6)	−0.0099 (6)
C10	0.0303 (7)	0.0564 (10)	0.0440 (8)	0.0047 (6)	0.0026 (6)	−0.0070 (7)
C11	0.0399 (8)	0.0494 (9)	0.0407 (8)	0.0069 (7)	0.0000 (6)	−0.0039 (7)
C12	0.0400 (7)	0.0403 (8)	0.0411 (8)	−0.0013 (6)	0.0052 (6)	−0.0112 (6)
C13	0.0376 (7)	0.0399 (8)	0.0523 (9)	0.0024 (6)	0.0137 (6)	−0.0112 (7)
C14	0.0417 (8)	0.0347 (8)	0.0518 (9)	0.0055 (6)	0.0109 (7)	−0.0084 (7)
C15	0.0535 (9)	0.0458 (9)	0.0534 (10)	−0.0032 (7)	0.0126 (8)	−0.0044 (7)

### 2-Methyl-4-[(4-methylphenyl)amino]benzoic acid Geometric parameters (Å, º)

**Table:**

O1—H1	0.8400	C7—H7A	0.9800
O1—C8	1.3184 (18)	C7—H7B	0.9800
O2—C8	1.2358 (18)	C7—H7C	0.9800
N1—H1A	0.8800	C9—C10	1.390 (2)
N1—C1	1.3800 (18)	C9—C14	1.394 (2)
N1—C9	1.406 (2)	C10—H10	0.9500
C1—C2	1.396 (2)	C10—C11	1.377 (2)
C1—C6	1.403 (2)	C11—H11	0.9500
C2—H2	0.9500	C11—C12	1.395 (2)
C2—C3	1.3812 (19)	C12—C13	1.392 (2)
C3—C4	1.4180 (19)	C12—C15	1.506 (2)
C3—C7	1.4977 (19)	C13—H13	0.9500
C4—C5	1.397 (2)	C13—C14	1.375 (2)
C4—C8	1.4715 (19)	C14—H14	0.9500
C5—H5	0.9500	C15—H15A	0.9800
C5—C6	1.379 (2)	C15—H15B	0.9800
C6—H6	0.9500	C15—H15C	0.9800
			
C8—O1—H1	109.5	O1—C8—C4	114.89 (13)
C1—N1—H1A	115.2	O2—C8—O1	121.59 (13)
C1—N1—C9	129.54 (13)	O2—C8—C4	123.47 (13)
C9—N1—H1A	115.2	C10—C9—N1	123.91 (13)
N1—C1—C2	117.79 (13)	C10—C9—C14	118.33 (14)
N1—C1—C6	124.10 (14)	C14—C9—N1	117.66 (14)
C2—C1—C6	118.07 (13)	C9—C10—H10	119.8
C1—C2—H2	118.3	C11—C10—C9	120.40 (13)
C3—C2—C1	123.46 (13)	C11—C10—H10	119.8
C3—C2—H2	118.3	C10—C11—H11	119.0
C2—C3—C4	118.02 (13)	C10—C11—C12	121.96 (15)
C2—C3—C7	118.33 (12)	C12—C11—H11	119.0
C4—C3—C7	123.65 (13)	C11—C12—C15	121.04 (15)
C3—C4—C8	122.02 (13)	C13—C12—C11	116.87 (14)
C5—C4—C3	118.50 (13)	C13—C12—C15	122.09 (14)
C5—C4—C8	119.34 (13)	C12—C13—H13	119.1
C4—C5—H5	118.6	C14—C13—C12	121.83 (13)
C6—C5—C4	122.70 (14)	C14—C13—H13	119.1
C6—C5—H5	118.6	C9—C14—H14	119.7
C1—C6—H6	120.4	C13—C14—C9	120.59 (15)
C5—C6—C1	119.22 (14)	C13—C14—H14	119.7
C5—C6—H6	120.4	C12—C15—H15A	109.5
C3—C7—H7A	109.5	C12—C15—H15B	109.5
C3—C7—H7B	109.5	C12—C15—H15C	109.5
C3—C7—H7C	109.5	H15A—C15—H15B	109.5
H7A—C7—H7B	109.5	H15A—C15—H15C	109.5
H7A—C7—H7C	109.5	H15B—C15—H15C	109.5
H7B—C7—H7C	109.5		
			
N1—C1—C2—C3	176.07 (13)	C5—C4—C8—O2	−168.92 (14)
N1—C1—C6—C5	−176.83 (14)	C6—C1—C2—C3	−1.5 (2)
N1—C9—C10—C11	−177.63 (14)	C7—C3—C4—C5	−178.68 (13)
N1—C9—C14—C13	177.01 (14)	C7—C3—C4—C8	5.6 (2)
C1—N1—C9—C10	−35.3 (2)	C8—C4—C5—C6	174.02 (13)
C1—N1—C9—C14	148.48 (16)	C9—N1—C1—C2	170.91 (14)
C1—C2—C3—C4	0.8 (2)	C9—N1—C1—C6	−11.6 (2)
C1—C2—C3—C7	−179.65 (13)	C9—C10—C11—C12	0.9 (2)
C2—C1—C6—C5	0.6 (2)	C10—C9—C14—C13	0.6 (2)
C2—C3—C4—C5	0.9 (2)	C10—C11—C12—C13	0.5 (2)
C2—C3—C4—C8	−174.82 (12)	C10—C11—C12—C15	−179.54 (14)
C3—C4—C5—C6	−1.8 (2)	C11—C12—C13—C14	−1.4 (2)
C3—C4—C8—O1	−175.73 (12)	C12—C13—C14—C9	0.9 (2)
C3—C4—C8—O2	6.7 (2)	C14—C9—C10—C11	−1.5 (2)
C4—C5—C6—C1	1.0 (2)	C15—C12—C13—C14	178.66 (14)
C5—C4—C8—O1	8.61 (19)		

### 2-Methyl-4-[(4-methylphenyl)amino]benzoic acid Hydrogen-bond geometry (Å, º)

**Table:**

*D*—H···*A*	*D*—H	H···*A*	*D*···*A*	*D*—H···*A*
O1—H1···O2^i^	0.84	1.81	2.6450 (16)	175
N1—H1*A*···*Cg*2^ii^	0.88	2.93	3.5460 (15)	128
C14—H14···O1^iii^	0.95	2.51	3.4368 (18)	165

Symmetry codes: (i) −*x*+1, −*y*+1, −*z*+2; (ii) −*x*+3/2, −*y*+1/2, −*z*+1; (iii) −*x*+3/2, *y*−1/2, −*z*+3/2.
